# The tip-of-the-tongue phenomenon in older adults with subjective memory complaints

**DOI:** 10.1371/journal.pone.0239327

**Published:** 2020-09-18

**Authors:** JungWan Kim, Minyoung Kim, Ji Hye Yoon

**Affiliations:** 1 Department of Speech and Language Pathology, College of Rehabilitation Sciences, Daegu University, Gyeongsan, Republic of Korea; 2 Division of Speech Pathology, Lee Rehabilitation Clinic, Incheon, Republic of Korea; 3 Division of Speech Pathology and Audiology, College of Natural Sciences, Hallym University, Chuncheon, Republic of Korea; 4 Research Institute of Audiology and Speech Pathology, Hallym University, Chuncheon, Republic of Korea; University of Glasgow, UNITED KINGDOM

## Abstract

In older adults with subjective memory complaints (SMCs), featuring a decline in memory but not exhibiting problems during medical examinations and objective memory tests, the weak links between nodes evident in the word retrieval process can be a primary factor for predicting mild cognitive impairment and dementia. This study examined the frequency of the “Tip-of-the-Tongue” (ToT) phenomenon according to age and subjective memory complaints of older adults, and identified differences in the resolution method using sequential cues. A celebrity naming task was performed on older adults (aged 50 to 79) with SMCs (n = 30) and without SMCs (n = 30), comparing the frequency of the ToT phenomenon and in resolution methods. We found that, even if our subjects with SMCs obtained normal results in the objective neuropsychology test, they experienced a significantly higher frequency of the ToT phenomenon than those without SMCs. In addition, subjects with SMCs showed a significantly lower rate of resolution, both spontaneous and following a syllabic cue, compared to those without SMCs. SMCs can be a very early marker of degenerative diseases causing cognitive dysfunction, and thus the selection of appropriate tools for early detection of SMCs is important. The proper naming task may sensitively detect subclinical symptoms of SMCs in subjects who are not classified as patients with cognitive impairments on general neuropsychological test. In addition, this task can identify weak connections between semantic and phonological nodes due to changes in the neural region of older adults with SMCs.

## 1. Introduction

With the increasing aging of society, interest in older adults has increased, and studies on patients with subjective memory complaints (SMCs) have become more common. Individuals with SMCs show a normal range of ability in objective memory tasks, but claim to have memory disorders. SMCs tend to increase with increasing age in the older adult population [1]. Among 747 persons who participated in the Korean Longitudinal Study on Health and Aging (KLoSHA) from 2005 to 2006, approximately 21.3% reported SMCs [2].

Recently, SMCs have become an important area of concern, as they have been found to correlate significantly with neuropathological changes observed in postmortem brain tissues, cognitive function decline, and dementia [3]. In a longitudinal study of the relationship between SMCs and objective cognitive impairment, dementia and cognitive dysfunction that appeared 1–7 years after the individuals experienced subjective memory impairment were found to be highly related to SMCs [4]. Consequently, SMCs can be a very early marker of degenerative diseases causing cognitive dysfunction, but can also represent a normal aging process or a secondary symptom arising from depression or psychological stress [5]. SMCs may be a clinically meaningful indicator of future cognitive decline, with individuals experiencing subjective memory complaints at increased risk of developing mild cognitive impairment (MCI) and dementia [6]. Subjects with SMCs have been reported to have 4 times higher risk of dementia as normal old adults, and SMCs have been shown to have a significant correlation with depression and anxiety [7]. Since SMCs are associated with neurodegenerative diseases in the long term, rapid discrimination of the high-risk group is extremely important for early detection and prevention. However, since these individuals often show normal performance in comprehensive neuropsychological tests [8], selection of a testing tool allowing better detection of the subtle changes related to SMCs is important.

One of the difficulties experienced by people who claim subjective memory problems is that “they cannot quickly come up with names.” This “Tip-of-the-Tongue” (ToT) phenomenon is a common type of speech error in which a person has a strong feeling of knowing the target word, but experiences retrieval failure, because of the inability to access phonological information [9, 10]. Older adults are known to experience the ToT phenomenon more frequently than young adults [10, 11], leading to reduced number of correct responses and increased verbal response time [12, 13]. Previous studies have examined the effects of age in the phonological encoding processes and have sought to verify whether the ToT phenomenon is a hallmark of old age, by comparing the frequency of ToT experiences between young and older subjects [9, 14]. In these studies, the ToT occurrence rate was reported to increase with increasing age. One of the causes of this increase is that older adults use less phonological information, as the connection for the transmission of phonological information is weaker in older adults than in young people. According to the transmission deficit model of the ToT phenomenon, these characteristics are regarded as being caused by weak connections among phonological-lexical representations [11]. Other previous studies have reported that the ToT phenomenon in older adults is related to a deficit in naming processes, including difficulties in accessing the phonological representation, rather than to a deficit in storage of semantic and phonological-lexical representations [9, 14].

There are several tasks for evaluating naming ability, including verbal fluency, confrontation naming, and picture description. Among these tasks, confrontation naming places relatively little demand on the higher cognitive linguistic process, which requires the effective manipulation of the semantic system via the integration of primary linguistic knowledge and cognitive and executive functions (e.g., working memory, planning ability, cognitive flexibility) [4, 5]. There are several psycholinguistic confrontation naming models, presenting slightly different points of views in terms of activation flow, but the conceptual components related to the naming processes are similar. Within these models, the stages of the naming process are as follows: (1) visual analysis and recognition of the stimulus, (2) access to the semantic representations, (3) access to the phonological–lexical representation, and (4) conversion of the phonological representation to a motor articulatory program for verbal expression. As confrontation naming is a task that requires access to the lexicon that matches the visual image of the stimuli, it is the most appropriate task for evaluating lexical retrieval. Diverse word categories (e.g., proper nouns and common nouns) can be used for confrontation naming, and tasks involving common nouns (e.g., the Boston Naming Test [BNT]) are widely used to investigate the naming deficit in patients with cognitive impairment, such as dementia. In terms of task difficulty, the process of naming proper nouns, such as buildings and celebrities, is more difficult and complex than that of naming common nouns [15]. In a comparative study on using noun categories with Alzheimer's disease patients, patients showed greater difficulties in naming proper nouns than common nouns [16]. Unlike common nouns, processing information related to proper nouns requires finding specific semantic information about individual persons/things and the lexical node representing semantic information of proper nouns is only connected to a single phonological node corresponding to the word. Accordingly, a naming task using proper nouns may allow a clear observation of the connections between semantic and phonological nodes.

In this context, the naming ability on proper nouns and the effect of phonemic cueing on the ToT phenomenon have been investigated to confirm the mechanisms underlying the ToT phenomenon in older adults [17]. This study demonstrated that aging affects the process of naming and the ToT phenomenon in terms of both incidence and resolution. In this previous study, when phonemic cueing was presented, both younger and older groups were able to resolve the ToT issue, but the extent of the effect was different. Unlike younger individuals, who could resolve the issue with the first syllable cue, older adults group only resolved the issue when provided with an additional last syllable cue, or by presenting a recognition processing task. This result indicates that the naming of proper nouns is more complicated than that of common nouns, and can be interpreted as showing that the vulnerabilities of this process are maximized as the node connection, or the brain activation, decrease due to aging [10, 11]. However, these results also indicated that the ToT phenomenon in older adults might be not a problem of storage, but may be due to weak links between phonological-lexical representations, because the cues were effective in facilitating the retrieval of target nouns. Therefore, phonologically related cues might enhance weakened phonological-lexical connections and consequently help resolve the ToT issue in older adults.

Oh and Ha [18] conducted a celebrity naming task for normal adults and MCI patients. When comparing the occurrence and resolution of the ToT phenomenon between the two groups, more ToT phenomena occurred in the MCI group. Studies examining the ToT phenomenon in SMCs are scarce, despite the increased recent attention. Therefore, by examining the effects of the ToT phenomenon and phonemic cues in patients with SMCs, and thus intermediate between normal adults and MCI, the phonological-lexical process depending on cognitive decline can be revealed. Furthermore, most studies on the ToT phenomenon in patients with neuro-linguistic impairment have been limited to the alphabetical written language system [11], and the findings of the study cannot be generalized to Korean subjects, because specific language systems and lexicons operate within different linguistic rules. In English, each letter within a syllable can be pronounced by itself. On the other hand, Korean consonant letters cannot be pronounced by themselves and are always and automatically accompanied by a vowel phoneme “—(/ɨ/).” When Korean examiners attempt to provide the single consonant phoneme “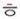 (/h/)” as in “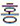 (hong/)” as a phonemic cue, they actually pronounce “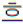 (/hɨ/).” Therefore, when examiners intend to provide a single phoneme “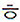 (/h/),” Korean subjects usually search for target words starting with the syllable “
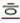
” rather than the single phoneme “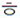.” For this reason, the Korean version of the BNT (K-BNT) usually provides a single syllabic cue rather than a single phonemic cue [19]. In addition, the number of syllables in proper nouns of people’s names is very diverse in different language systems; thus, the portion of the single phonemic/syllabic cue may vary depending on the number of whole syllables. For example, the portion of phonemic/syllabic cueing in the full names “Abraham Lincoln” and “Pearl Buck” is different. In Korean, however, personal names generally consist of a surname of one syllable and a given name of two syllables, which makes it easy to control the portion of the syllabic cueing for proper nouns.

This study examined the frequency of the ToT phenomenon according to age and SMCs of older Korean adults and identified differences among the subject groups in the resolution method using sequential cues. This study attempted to verify whether weak connections between semantic and phonological nodes are observed due to changes in the neural regions of older adults with SMCs.

## 2. Methods

### 2.1. Participants

The G*power 3.1.2 software program was used to estimate the minimum sample size following the procedures described by Faul et al [20]. Hence, a total of 68 participants (34 participants per group) were needed for an effect size of d = .25, power = .8, and p-value = .05. Sixty eight community-dwelling older adults aged between 50 and 79 years residing in South Korea were recruited from August to October 2018. All participants were subjected to pre-evaluation questions in order to screen their hearing and vision. The participants had no history of neurological or psychiatric disorders. All participants were administered the following tests in order to more fully assess the characteristics of the population sample: (a) Mini-Mental Status Examination (MMSE) [21], (b) Short-form Geriatric Depression Scale (SGDS) [22], and (c) SMC questionnaire. Regarding the SMC questionnaire, there is no single standard for the diagnosis of SMCs. Therefore, the subjects satisfying the following criteria were classified as SMCs, based on the classification criteria used in previous studies [23–26]: (a) absence of structural or functional brain problems or psychiatric disease that could account for any memory impairment; (b) the subject consistently claims subjective memory impairment for 6 months or longer; (c) the subject believes that their current memory is worse in comparison to 5–10 years ago; (d) the subject can provide a lively example of memory problems that occur during everyday life; (e) memory impairment experienced at least once a week; and (f) normal performance during the objective neuro-psychology test on memory. According to the test results, 8 subjects that did not meet normal limits in the MMSE [21] and SGDS [22] were excluded. The 60 participants were divided into two groups (30 older adults with SMCs and 30 without SMCs). There were no significant differences between older adults with and without SMCs with regard to age, level of education, MMSE score, and SGDS (all p>.05; see Table 1). This research was approved by the Institutional Review Board of Daegu University (IRB#: 1040621-201807-HR-010-02).

**Table 1:** Subjective characteristics.

Group	OA-w/o SMCs (n = 30)	SMCs (n = 30)
Age	64.10(±4.53)	68.50(±4.76)
Level of education	6.90(±3.47)	6.50(±3.99)
MMSE	28.22(±2.23)	27.49(±1.82)
SGDS	1.20(±1.82)	1.83(±2.36)

OA-w/o SMCs: older adults without subjective memory complaints, SMCs: subjective memory complaints, MMSE = Mini-Mental Status Examination, SGDS = Short-form Geriatric Depression Scale (cut-off score:8)

### 2.2. Materials

The method used in previous studies to select words to induce the ToT phenomenon was applied [11]. Among proper nouns, names of people who were reported to show the lowest production accuracy in normal older adults were used to create the celebrity naming task. Among 156 celebrities selected according to frequency of reports on the usage frequency of modern Korean words, 60 Korean celebrities were selected [27]. In general, Korean names consist of a surname of 1 syllable and a given name of 2 syllables, for a total of 3 syllables. Since two- or 4-syllable names are rare, only 3-syllable names were used. To check whether the words selected according to objective frequency were similar to the subjective frequency of subjects, celebrity naming was carried out on normal adults aged between 28 and 75 years by measuring their subjective frequency on a 5-point Likert scale (1: none, 2: once a year, 3: once a month, 4: once a week, 5: once a day or more). Based on the results of the Likert scale, 50 celebrities who showed a subjective frequency score of 3.0 or above were selected. Photos of the celebrities used in this study were selected according to the following criteria: (a) The photo must be sufficiently clear to identify the celebrity; (b) there must be only one celebrity naming response for each photo; (c) there must be no cues such as clothes and accessories useful to identify the celebrity; and (d) the celebrity must be identified regardless of the time period. After showing photos of the celebrities to 20 normal adults (mean age: 48.5 years), 10 photos that showed incorrect responses (i.e., do not know, wrong naming, or ToT phenomenon) were excluded. Thus, a total of 40 photos that showed 100% correct responses were selected. The random number table on Microsoft Office Excel 2018 was used to randomize the order of the photos, and the order was rearranged so that 3 or more celebrities in the same time period (= coexistent in time) would not appear consecutively.

### 2.3. Procedures

**Fig 1:**
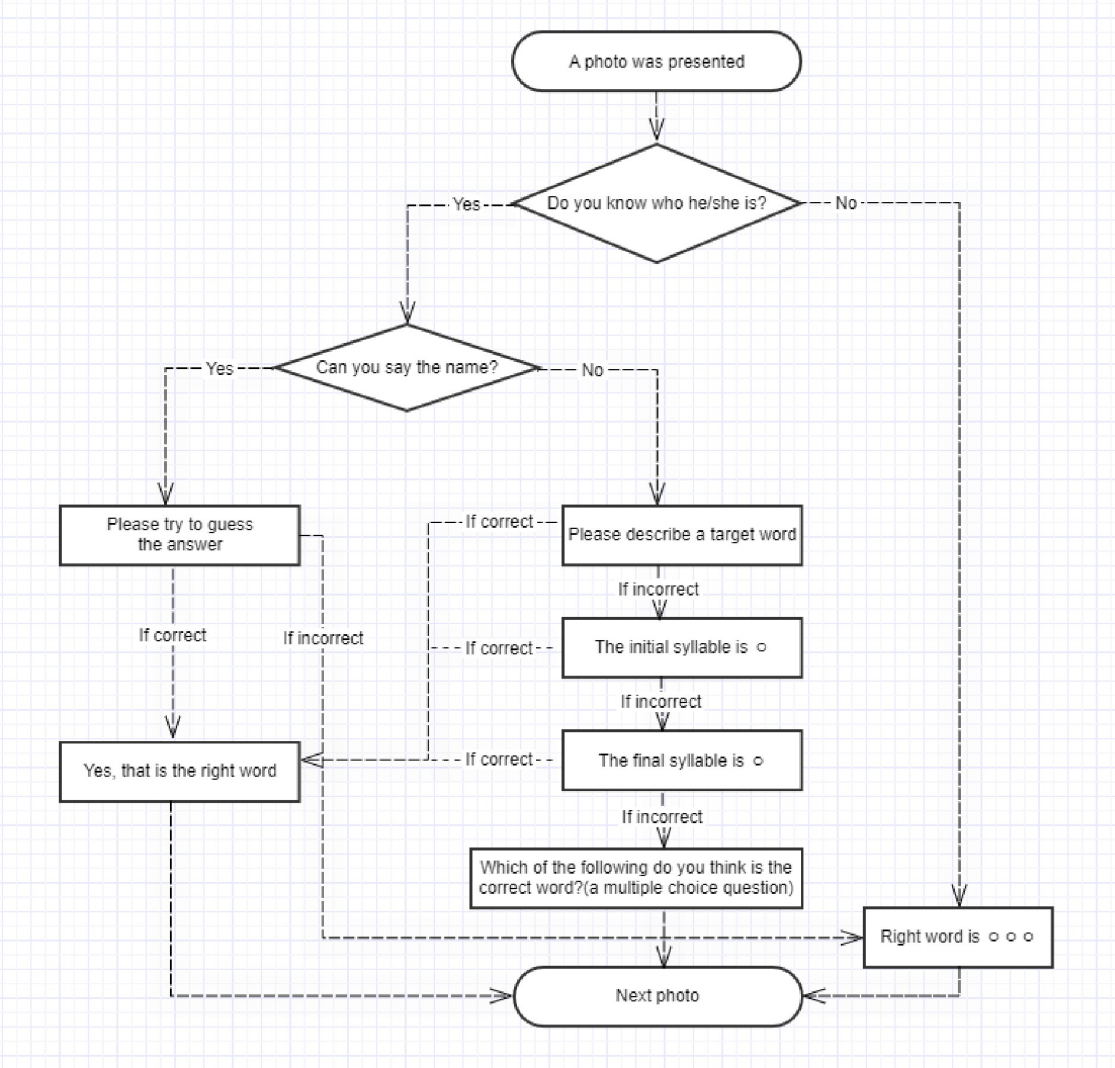
Sequence of questions for inducing ToT phenomenon.

To determine the occurrence rate (frequency) and resolution method of the ToT phenomenon experienced during the naming task, each participant completed the proper noun naming task. The task was carried out in a one-on-one session between the researcher and subject in a noise-controlled environment. After presenting the definition of the ToT phenomenon, the overall experimental process was explained. Then, 3 practice questions were provided so that subjects could adequately understand the experimental process. The procedure of the task is as follows (Fig 1): (a) The subject looked at the photo presented and reported one state among ‘knowing who it is’, ‘knowing who it is but being unable to immediately come up with name (ToT state)’, or ‘not knowing who it is.’ (b) If the subject reported ‘knowing who it is’, the subject was told to say the celebrity’s name within 15 seconds, which was regarded as a correct response. (c) If the subject reported the ToT state, the subject was asked to explain the semantic information about the target word. If the subject said the target word during this process (self-semantic cue), it was considered a ‘spontaneous resolution’ and the subsequent photo was shown. (d) If the ToT state continued after producing semantic information about the target word, the researcher sequentially provided the first syllable and last syllable of the target word to the subject. If the subject said the target word during this process, it was considered a ‘resolution after the syllabic cue.’ (e) If the ToT phenomenon could not be resolved with the syllabic cue, the researcher gave 4 options, including the target word, to the subject for the word recognition task. This confirmed whether the subject potentially had the ToT phenomenon for the target word or possessed incorrect knowledge regarding the celebrity’s name. All responses were recorded with the subjects’ consent.

### 2.4. Scoring

(1) Mean ToT Frequency (%)

This is defined as the ratio of cases in which spontaneous resolution did not occur after showing the photo for 15 seconds (ToT phenomenon). However, cases of partial word retrieval were excluded when calculating the ToT frequency.

(ToT frequency / overall naming frequency–partially retrieved word frequency) x 100

(2) Method of Resolution (%)

The resolution rate of the ToT phenomenon was analyzed only with the words that showed the ToT phenomenon. There were two resolution methods, as presented below.

*Spontaneous resolution (pop-ups only, %)*.

(Frequency of the ToT phenomenon resolved spontaneously / overall ToT frequency) x 100

*Resolutions after syllabic cues (%)*.

(Frequency of ToT resolution after the syllabic cue / frequency of unresolved ToT phenomenon after self-semantic cue) x100

### 2.5. Reliability

Record sheets of 15 subjects corresponding to 25% of the complete measurement data were randomly taken, and reliability was calculated by comparing the analysis results between the first and second evaluators. Reliability between the evaluators was found to be 97.5%.

### 2.6. Statistical analyses

The Welfare of Senior Citizens Act in Korea defines senior citizens to be persons aged 65 or older, and various neuropsychological tests show different results in different age groups. Thus, subjects were divided into two age groups (50–64 years and 65+ years). We examined the mean ToT frequency (%) according to the subject group as a between-subject design and according to seniority (50–64 years, 65+ years) as a within-subject design, to evaluate the main and interaction effects of these two variables. Variables that showed significant differences in this analysis were included in post-hoc analyses for specific comparison. In addition, repeated measures ANOVA was performed to assess the differences in the ToT phenomenon resolution method between the groups with and without SMCs.

## 3. Results

When assessing the mean difference in ToT frequency according to age between the groups with and without SMCs (Table 2), the main effect of the group was significant (F = 42.827, df = 1, MS = 5191.260, *p* < .001), but the main effect of age was not (F = .239, df = 1, MS = 28.981, *p* = .627). There was no interaction effect between the group and age variables (F = .003, df = 1, MS = .338, *p* = .958). As part of the post hoc test, a t-test was performed to confirm the average difference in ToT frequency (%) and the effect size between the groups with and without SMCs. Older adults without SMCs showed significantly lower ToT frequency (%) than older adults with SMCs (*t*(_***58***_) = -6.646, p < .001, Cohen's D = 10.8416).

**Table 2:** Mean ToT frequency (%) by group and age variables.

Group	50~64 years	65+ years	total
OA-w/o SMCs (n = 30)	12.16%(±7.37)	13.70%(±8.18)	12.93%(±7.69)
SMCs (n = 30)	30.91%(±12.17)	32.15%(±14.68)	31.53%(±13.26)

OA-w/o SMCs: older adults without subjective memory complaints, SMCs: subjective memory complaints

**Fig 2:**
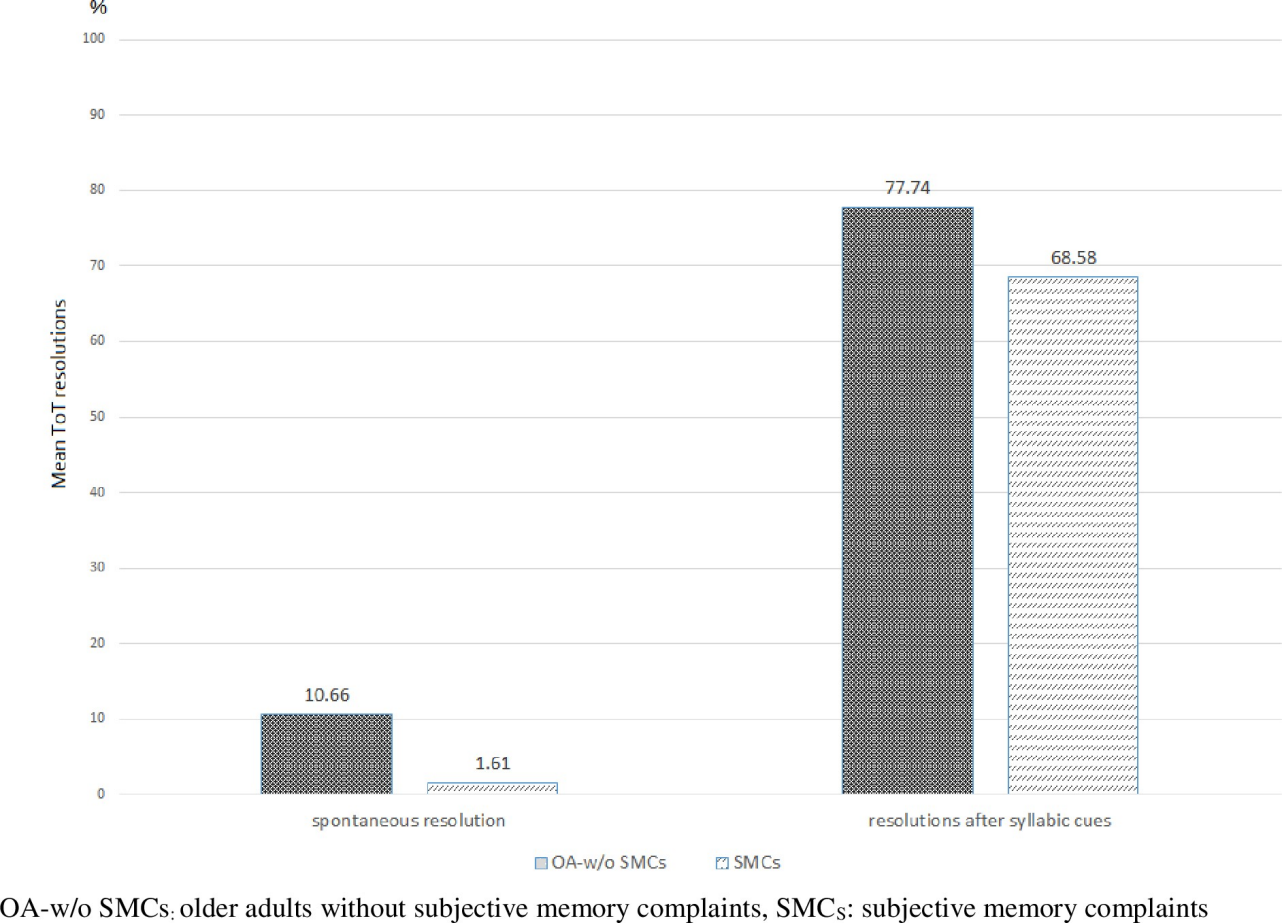
Mean ToT resolutions (%) by group and type of resolution.

Since age was not identified as significant in the two-way ANOVA analysis, we performed repeated measures ANOVA to assess the difference in the ToT phenomenon resolution method between the groups with and without SMCs. The main effect of the group was significant (F = 5.16, df = 1, MS = 2260.28, *p* < .05) and the effect of the resolution method within the groups was significant (F = 478.87, df = 1, MS = 122511.34, *p* < .001). The interaction effect between group and resolution method was not significant (F = .000, df = 1, MS = 0.08, *p* = .758), as illustrated with greater detail in Fig 2. ANOVA results are presented in Table 3.

**Table 3:** Mean ToT resolutions (%) by type of resolution and group.

	OA-w/o SMCs (n = 30)	SMC_*S*_ (n = 30)
spontaneous resolution	10.66%(±21.33)	1.61%(±4.41)
resolutions after syllabic cues	77.74%(±24.98)	68.58%(±18.87)

OA-w/o SMCs: older adults without subjective memory complaints, SMC_***S***_: subjective memory complaints

## 4. Discussion

This study examined differences in ToT frequency and resolution methods according to age and SMC_***S***_ in older adults aged between 50 and 79 years. Even if our subjects with SMCs obtained normal results in the objective neuro-psychology test, those with SMCs experienced a significantly higher frequency of ToT phenomena than those without SMCs. Interestingly, there was no significant difference in ToT frequency according to age group. Rather, the ToT frequency in subjects with SMCs aged 50–64 years was higher than that of those without SMCs, regardless of age. In other words, the ToT phenomenon was influenced more by subjective memory problems than by aging.

So far, the incomplete activation hypothesis [28] and the transmission deficit hypothesis [11] have been posited to explain the ToT phenomenon. The incomplete activation hypothesis proposes that word production fails because the connection between a word’s meaning (semantics) and its form (phonological representations) cannot be activated and/or is activated only in part. According to the transmission deficit hypothesis, a lexical node in the semantics-associated region becomes activated, but some phonological information remains inaccessible, due to insufficient priming across critical connections required for retrieving the target word. In order to produce proper nouns, the subject needs to activate a more specific and precise route, without help from other networks. From these points of view, older adults with SMCs may have reduced ability to activate appropriate and accurate information or to transmit information at each stage in the production of proper nouns. Therefore, despite the relatively young age of the older adults with SMCs, it is possible that these individuals are more susceptible to such deterioration of function than older individuals without SMCs, and may experience the ToT phenomenon more frequently. Our findings also indicate that weak and incomplete activation/transmission of information among semantic, lexical, and phonological nodes in the naming task were present in the SMCs group.

We attempt to explain this phenomenon by considering the underlying mechanism of the naming process as unique for proper nouns. To understand the ToT phenomenon as related to proper nouns, we reviewed a functional model for facial recognition. According to Bruce and Young’s model [29], the visual attributes of the face are analyzed through the visual structural encoding process. After the visual analysis, visual information accesses previously stored information for the face held in the face-recognition units. The next stage is the person identification node, which involves a one-person identity node for each person. In this stage, identity-specific semantic codes are accessed from the person identity nodes, and subsequently, name codes are retrieved. During this last stage, the target name codes (e.g., lexical information) are transformed into the corresponding phonological information for the name. In the process of proper nouns, lexical nodes that represent semantic information are uniquely associated with a single semantic node to which that word refers [11]. If the connection of semantic nodes is weakened, it is impossible to transfer information, because there is no connection with other semantic nodes that can be replaced when insufficient priming occurs.

Another explanation for the ToT phenomenon is that the difficulty of the task, which affects cognitive processing, may have affected the naming performance. The process of naming proper nouns is considered more complicated than the process of naming other nouns [30, 31]. Individuals with SMCs may be more affected by the cognitive burden, due to lack of subjective or objective cognitive ability, than those without SMCs. Therefore, it is possible that individuals with SMCs showed lower performance in the complex naming task for difficult nouns. In addition, word retrieval is not an all-or-none process. Partial information about the target name is quite commonly available when the target itself is not retrieved. However, individuals with SMCs showed a significantly lower rate of spontaneous resolution and resolution following a syllabic cue than those without SMCs. The rate of ToT resolutions of the SMCs group increased after receiving a syllabic cue, but was still significantly lower than that of the group without SMCs. A previous study has examined the degree of resolution by presenting the prime stimulus before computing the target word [9]. The ToT phenomenon was mostly reported to show spontaneous resolution by “pop-up” [10]. To obtain help from the cue, mental flexibility and the ability to associate cues with previously stored information are used while implementing the given cue. Some studies have shown that frontal lobe functions, such as generation ability and memory strategy, are decreased in individuals with SMCs [32]. The generation ability and memory strategy also affect ToT resolutions and may be a hallmark of individuals with SMCs [33].

Interestingly, we observed that the effect of the cue differed depending on the type of cue. When examining the degree of help according to the type of cue, both individuals with and without SMC_***S***_ tended to find syllabic cues more helpful than self-semantic cues, with a 68.58–77.74% rate of correct responses when a syllabic cue was provided. The explanation for this phenomenon could be two-fold. First, it can be explained by the difference in the degree of information contained in the cue. A phonemic/syllabic cue is more direct than a semantic one. In reviewing the cue presentation order in language interventions for naming disorders [34, 35], it has been reported that the ranking of cue types by decreasing content of direct information is the following: modeling, syllabic cue, phonemic cue, semantic cue, and re-asking. The second explanation for the effect of syllabic cues has to do with the characteristics of Korean proper nouns. According to the naming models [29], when the target proper noun is retrieved, competing alternatives in the phonological output lexicon might be also activated. Since Korean names usually consist of three syllables, giving the first and last syllable cues provides more than 60% of the total information. Activation at the phonological-lexical representation level by syllabic cues may either inhibit the activation of competing alternatives that do not fit the syllable, or select the appropriate proper noun among alternatives. Therefore, syllabic cues, by providing more direct information, might have played an important role in accessing the mental lexicon of proper nouns in Korean language by simplifying the naming process. In the previous study [19], even in the clinical dementia rating 2 group of Alzheimer’s disease (AD), considered a moderate cognitive disorder, Korean syllabic cues significantly facilitated the correct responses.

On the other hand, individuals with SMCs found little assistance from a semantic cue that was derived from the target words. The semantic cue helped to resolve about 10% of ToT occurrences in individuals without SMCs, but only about 1% in those with SMCs. The minimal effect of the semantic cue on individuals with SMCs may be explained through the confrontation naming model. It has been reported that individuals with SMCs may experience the ToT phenomenon not because of a problem at the semantic stage itself, but because of an issue in the connection between the lexical and phonological nodes. In the naming models, after analyzing the visual information, the semantics and lexical nodes associated with the visual information should be accessed, and finally the phonological information should be retrieved. Semantic cues would provide more direct assistance to semantic or lexical stages, which are earlier levels than phonological–lexical stages. Individuals with mild to moderate AD, whose semantic storage is known to be compromised, were aided by presentation of a semantic cue when experiencing difficulty finding words in the BNT [19]. In summary, patients with AD were aided by semantic and syllabic cues due to the deficits of semantic and phonological-lexical stages, respectively. Compared with the previous study [19], our results might reflect the possibility that individuals with SMCs did not gain much from the self-semantic cue in resolving the ToT phenomenon. In addition, our result suggests that, in individuals with SMCs, the ToT phenomenon reflects a problem at the later stages (phonological–lexical stages), rather than at the semantic stage, in the word-retrieval models.

The present study empirically confirmed the occurrence of the ToT phenomenon and its resolution patterns, which may occur during the pathological aging process. The ToT phenomenon occurred more frequently in individuals with SMCs than in individuals without SMCs, and the ToT resolution rate in individuals with SMCs was lower than in individuals without SMCs, even after the provision of cues. This means that weak connections among phonological-lexical representations occur during the word retrieval stage in individuals with SMCs. This also suggests that SMCs can be considered a factor affecting the ToT phenomenon, and that its resolution depends on factors other than the age or subtypes of cue. In terms of clinical implications, the appropriate naming task may sensitively detect subclinical symptoms of SMCs in individuals who are not classified as patients with cognitive impairments on a general neuropsychological test. Such tasks can effectively indicate the ability to select proper names that have a small number of links and nodes representing semantic information in the early stages of SMCs. Therefore, observation of the ToT phenomenon during a proper noun naming task can help to detect cognitive difficulties related to SMCs in the early stages. Furthermore, early intervention can be applied for cases with mild cognitive impairment and dementia with identified naming difficulties related to SMCs in the early stages, by using a task that includes word types involving a small number of nodes and connected links. From the language-specific point of view, this study has further significance in that it provided syllable cues suitable for the Korean language system and confirmed their effect.

The limitations of this study were as follows. The task of naming celebrities by looking at their photos is part of the naming task for evaluating the ToT phenomenon, and there are limits in generalizing this task to the more general naming difficulties of subjects with SMCs. Since performance in a naming task differs according to word frequency and word category [11], future studies should implement diverse naming tasks according to word frequency and word category and confirm the distinct aspects observed in subjects with SMCs in order to explain in greater depth the difficulties in the word retrieval process present in the preclinical stage. The number of pictures selected in this study was not high enough to allow sufficient variability in the effect of SMCs. Therefore, the results should be interpreted with caution, and a larger number of stimuli need to be applied in future studies. According to previous studies, individuals with SMCs should be classified into sub-groups according to the influencing variables [8], as subjective memory problems in older adults can have various causes, such as depression, perfectionist inclination, and memory problems. Accordingly, it should be possible to reveal relevant information about memory impairment by examining the ToT phenomenon in SMC subgroups. The higher ToT frequency shown by subjects with SMCs compared with those without SMCs regardless of age suggests that subjective memory problems have a greater effect on the ToT phenomenon than age. However, group comparisons have limited power to solve this issue when the sample size is small. Therefore, future studies with large sample sizes are needed to identify the factors affecting the ToT phenomenon.
